# A Robust Method for Simultaneous Determination and Risk Assessment of Multiresidual Pesticides in Fishery Products

**DOI:** 10.3390/toxics12090633

**Published:** 2024-08-28

**Authors:** Myungheon Kim, Tae-hwa Kim, Jong-Woo Park, Yoonmi Lee, Mi-Ra Jo, Yong-Sun Moon, Moo-Hyeog Im

**Affiliations:** 1 Department of Food Engineering, Daegu University, Gyeongsan 38453, Republic of Korea; 2 Analysis Technology and Tomorrow, Daegu 42703, Republic of Korea; 3 Food Safety and Processing Research Division, National Institute Fisheries Science, Busan 46083, Republic of Korea; 4 Department of Horticulture and Life Science, Yeungnam University, Gyeongsan 38541, Republic of Korea

**Keywords:** pesticide analysis, method validation, fishery product, monitoring, risk assessment

## Abstract

In this study, we developed and validated a multiresidue analytical method for the simultaneous detection of 24 pesticides in fishery products. Using the EN15662 extraction method and C_18_ as the adsorbent for purification, the validation results complied with Codex guidelines, achieving recovery rates between 70% and 120% and relative standard deviation values (%RSD) within 20%, indicating excellent performance. The limit of detection ranged from 0.25 to 0.8 ng/kg, and the limit of quantification was between 3 and 10 ng/g, providing sufficient sensitivity to comply with future regulatory standards. The calibration curves for all 24 pesticides exhibited great linearity (R^2^ > 0.98), also satisfying the Codex requirements. The matrix effect was less than 30% for some pesticides—within ±20%—indicating minimal interference from impurities. An analysis of 300 fishery samples from nine regions across South Korea detected lufenuron at 10 ng/g in eels; however, the risk assessment was below 0.19%, posing no significant hazard to public health. This newly developed analytical method proved effective for the multi-analysis of pesticide residues in fishery products, offering rapid and reliable monitoring of the import and export safety of fishery products.

## 1. Introduction

The need for alternative protein sources has increased owing to the limited productivity of livestock and the resulting shortage of protein supply. With the increase in the production of cultured fish as an alternative protein source, fishery products have become a significant component of the global food industry. Fish consumption is recommended for its high content of omega-3 fatty acids, proteins, polyunsaturated fatty acids, and vitamins, which together help prevent various diseases, including cardiovascular disorders [1,2,3,4]. According to the Food and Agriculture Organization (FAO) of the United Nations, South Korea ranks within the top 3% of 239 countries worldwide for fishery product consumption [1,5]. Fishery production in South Korea is classified into four categories: marine aquaculture, coastal fisheries, deep-sea fisheries, and inland fisheries [6]. To produce high-quality fishery products, drugs are used in fish farming for promoting growth and preventing or treating diseases [7,8]. However, pesticides used in nearby agricultural areas or golf courses can contaminate surrounding rivers, reservoirs, and lakes, leading to unintended fish contamination [9,10]. Since most pesticides are toxic synthetic organic compounds, the consumption of food contaminated due to inadequate pesticide residue management can harm human health [11,12,13,14].

Organochlorine pesticides have been globally restricted since the early 1970s owing to their potential to cause congenital malformations, reproductive and endocrine disorders, immune system dysfunction, and cancer [15]. These compounds are structurally stable, resistant to degradation, exhibit long half-lives, and display high octanol–water partition coefficients, which together lead to bioaccumulation in the muscles and fat tissues of aquatic organisms, resulting in chronic toxicity in humans through long-term exposure [12,16,17]. Consequently, systematic management of aquatic environments and fish farms, which requires effective analytical methods for monitoring the presence of pesticide residues in these ecosystems, is essential to prevent pesticide residue contamination [18].

Worldwide, South Korea, along with the United States, Australia, the European Union, and Japan, have established pesticide maximum residue levels (MRLs) for domestic and imported fishery products to ensure food safety. Additionally, the Codex Alimentarius Commission (Codex) has set international standards to ensure fairness in global food trade.

Regarding pesticide management in food, the positive list system (PLS) was implemented for all agricultural products beginning 1 January 2019, applying a limit of detection (LOD) of 0.01 mg/kg for food products for which no pesticide MRLs have been established. Since 1 January 2024, the PLS of veterinary medicines has been applied to livestock products, including cattle, pigs, chickens, milk, and eggs. Following this trend, the PLS of pesticides can also be applied to fishery products.

Various analytical methods, such as solid–liquid extraction, Soxhlet extraction, sonication-assisted extraction, solid-phase extraction, and accelerated solvent extraction related to gel permeation chromatography, have been introduced [19,20,21]. However, these methods are often expensive, labor intensive, and time consuming; furthermore, they show low sample throughput and require advanced analytical expertise and expensive specialized equipment [22]. To address these issues, the quick, easy, cheap, effective, rugged, and safe (QuEChERS) method was developed, and it has been improved to suit various compounds and matrices [23,24,25]. Recent research includes a study on pesticide residue analysis in tilapia using the QuEChERS method [26], showing an improved multiresidue analysis method for 32 pesticides in aquaculture fish using acetonitrile with 1% formic acid and acetonitrile/methanol (5:5 *v*/*v*), with 1% formic acid as the extraction solvent [27]. Another study compared three extraction and purification methods for shrimp and eel [28]. However, most studies have focused on a single fish species, with limited research on methods that can simultaneously analyze multiple types of fishery products.

In this study, we aimed to develop an effective analytical method for the simultaneous analysis of 24 pesticides in fishery products, including triazines, triazoles, amides, carbamates, neonicotinoids, and anilines, by referencing and modifying the QuEChERS method. The new method was successfully applied to various types of fishery products, followed by comprehensive monitoring and risk assessment.

## 2. Materials and Methods

### 2.1. Reagents

The 24 pesticides analyzed in this study (acetamiprid, azimsulfuron, azinphos-methyl, azoxystrobin, carbendazim, carbofuran, clothianidin, daimuron, dichlorvos, difenoconazole, dinotefuran, diuron, fenobucarb, flubendiamide, hexaconazole, indoxacarb, lufenuron, pyrimisulfan, tebufenozide, terbuthylazine, thiacloprid, thiamethoxam, tricyclazole, and trifloxystrobin) were purchased from Kemidas (Gunpo, Republic of Korea). Acetonitrile (HPLC-grade) was obtained from J. T. Baker (Center Valley, PA, USA), and acetic acid (99%) was purchased from Duksan Chemicals (Ansan, Republic of Korea). Magnesium sulfate anhydrous (MgSO_4_; 99.5%) and sodium chloride (NaCl; 99.5%) were acquired from Junsei (Tokyo, Japan), and sodium citrate dihydrate (Na_3_Cit∙1.5H_2_O; ≥99.0) and sodium acetate (NaOAc; ≥99.0) were purchased from Sigma-Aldrich (St. Louis, MO, USA). Primary secondary amine (PSA) and C18 were acquired from Agilent Technology (Santa Clara, CA, USA).

### 2.2. Sample Preparation

Fishery product samples were purchased from fish farms in nine regions across the country: Chungcheongbuk-do, Chungcheongnam-do, Jeju-do, Jeollabuk-do, Jeollanam-do, Gangwon-do, Gyeonggi-do, Gyeongsangbuk-do, and Gyeongsangnam-do. Following the Korean Food Code, 300 samples were collected, including 20 samples each of 13 fish species (carp, Chinese muddy loach, crucian carp, eel, far eastern catfish, flathead mullet, Korean rockfish, mirror carp, olive flounder, rainbow trout, red sea bream, sea bass, starry flounder), one crustacean species (whiteleg shrimp), and one shellfish species (abalone) [29]. Crustaceans and shellfish were shelled and eviscerated, and fish samples were prepared by removing fins, bones, and heads, followed by homogenization of the edible parts, including the skin, with dry ice using a mixer (Grinmic gold-DA10000G, Daesung Artlon, Paju, Republic of Korea). All samples were stored at −20 °C until analysis.

### 2.3. Optimization of Analytical Methods

We used eel, chosen for its high fat content, as a control sample, treating it with 10 ng/g of a mixed pesticide standard. The liquid–liquid partitioning efficiencies of the EN15662 and AOAC methods were compared to establish the extraction method [30]. The EN15662 method used 20 mL of acetonitrile (+0.1% acetic acid), 4 g of MgSO_4_, 1 g of NaCl, 1.5 g of Na_3_Cit∙1.5H_2_O, and 5 mL of water. The AOAC method used 20 mL of acetonitrile (+0.1% acetic acid), 6 g of MgSO_4_, and 1.5 g of NaOAc. For purification, the efficacy of 50 mg of PSA, combined with 50 mg of C_18_, was compared to that of 50 mg of C_18_ alone. The optimal analytical method was determined based on the results of the recovery tests.

### 2.4. LC–MS/MS Analytical Conditions

Pesticide residues in fishery products were analyzed using an LC–MS/MS system (LCMS8060NX, Shimadzu, Kyoto, Japan), and data were processed using Labsolution SP2 software (version 5.75) (LCMS8060NX, Shimadzu, Kyoto, Japan). LC was performed using a C18 column (2.1 mm × 150 mm, 3.0 μm, Shimadzu Shim-pack GIST-HP) (Kyoto, Japan). Two mobile phases, A (aqueous, 0.1% formic acid, and 5 mM ammonium formate) and B (methanol, 0.1% formic acid, and 5 mM ammonium formate), were used at a flow rate of 0.2 mL/min. The gradient started at 5% B for 1 min, increased to 60% B over 2 min, then to 100% B within 10 min, and was maintained for 3 min. Finally, it was returned to 5% B over 0.1 min and held for 3.9 min, for a total of 20 min. MS/MS was conducted in the positive electron spray ionization mode with an interface voltage of 4.0 kV (Table 1). Multiple reaction monitoring conditions were established for each of the 24 pesticides by identifying the precursor ions and scanning for product ions. Based on the sensitivity and selectivity, at least two product ions for each pesticide were selected for quantitative and qualitative analyses, and their optimal collision energies were determined.

### 2.5. Method Validation

The newly developed analytical method presented in this study was validated according the Codex (2023) guidelines [31], assessing linearity, LOD, limit of quantitation (LOQ), accuracy, and precision. Olive flounder, eel, abalone, and whiteleg shrimp with no pesticide residues were used as control samples. Linearity was determined using matrix-matched calibration curves, with calculation of the coefficient of determination (R^2^). LOD and LOQ were determined by analyzing pesticide-free samples and calculating the signal-to-noise (S/N) ratio of the chromatographic peaks. LOD was defined as the concentration with an S/N ratio of 3 or higher, and LOQ was defined as that with an S/N ratio of 10 or higher. Recovery tests were conducted to assess accuracy and precision by spiking the control samples at the LOQ, 10 times the LOQ, and 50 times the LOQ with a mixed standard solution, followed by five replicate analyses.

### 2.6. Risk Assessment

The risk assessment was conducted based on the method reported by Kim et al. [32]. Estimated daily intake (EDI) for the detected pesticides and fishery products consumption data were calculated using nine different scenarios (Equation (1)), followed by exposure assessment.

EDI (ng/person/day) = DFI ^a^ (g/person/day) (1, 2, 3) × DPC ^b^ (ng/g) (A, B, C) (1)

where ^a^—daily food intake, ^b^—detected pesticide concentration

1—average intake by fish species,

2—extreme (99th percentile) intake by detected fish,

3—extreme (99th percentile) intake by fish species,

A—(detected pesticide + LOQ value for non-detection)/number of test (20),

B—detected pesticide/number of detections, and

C—maximum detected pesticide.

Consumption data were based on the Korea National Health and Nutrition Examination Survey (KNHANES) for 2017–2021 [33], considering the average and extreme (99th percentile) intake of fish and shellfish (Table 2). For species with no available consumption data, intake values were estimated as half the minimum mean and extreme (99th percentile) intake of similar species. The average body weight of Koreans was assumed to be 60 kg. The health risk index (%ADI) was calculated by comparing the EDI with the acceptable daily intake (ADI) and expressing it as a percentage of ADI (Equation (2)).

(2) $$\%\mathrm{ADI}=\frac{\mathrm{EDI}\;\left(\mathrm{ng}/\mathrm{person}/\mathrm{day}\right)}{\mathrm{ADI}\;\left(\mathrm{ng}/\mathrm{person}/\mathrm{day}\right)}\times 100$$

### 2.7. Statistical Analysis

Statistical analyses were performed using Excel 2013 (Microsoft Corporation, Seattle, WA, USA). Data are presented as the mean and standard deviation, resulting from the analysis of five replicates.

## 3. Results and Discussion

### 3.1. Specificity

**Figure 1:**
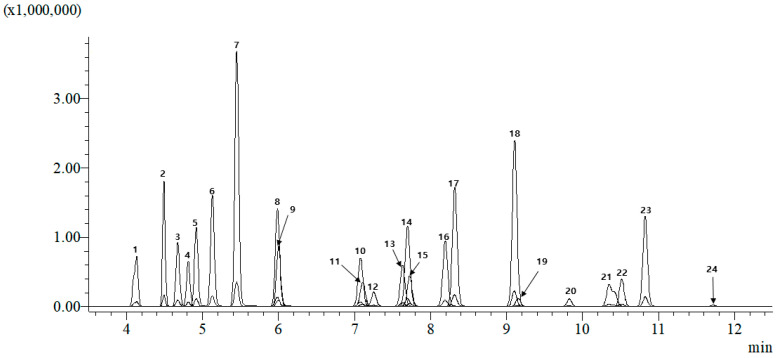
Total ion chromatogram (TIC) of a 100 ng/g mixture of 24 pesticides in solvent. The chromatogram displays the separation of 24 pesticide compounds in a solvent using LC–MS/MS, demonstrating their distinct retention times and ionization profiles. The numbers of the peaks correspond to the sequence listed in Table 1.

The total ion chromatograms (TIC) of the 24 pesticides analyzed are shown in Figure 1. The qualitative and quantitative ions of these pesticides were sufficiently separated, allowing for distinct identification based on their retention times. Pesticides with similar retention times were differentiated by their *m*/*z* values for precursor and product ions.

### 3.2. Establishment of Sample Extraction and Purification Methods

The efficiency of pesticide extraction from fishery products was evaluated by comparing the EN15662 [34] and AOAC [30] methods. Additionally, to assess the effectiveness of removing interfering substances, such as fats, during purification, two purification approaches were compared: the use of C18 (50 mg) alone and a combination of PSA (50 mg) and C18 (50 mg). Table 3 summarizes the extraction and purification effectiveness results.

The EN15662 method demonstrated recoveries above 70% for all 24 pesticides, whereas the AOAC method resulted in recoveries below 60% for 8 pesticides, including carbendazimhad. Kwak et al. [35] and Lehotay et al. [36] reported that the acetate buffer in the AOAC method, which lowers sample pH, hampered pesticide transfer to acetonitrile, reducing recovery rates. Additionally, the EN15662 method showed a lower matrix effect than the AOAC method. Consequently, the EN15662 method, which demonstrated superior recoveries, was selected for the extraction. Since the amine groups of the PSA adsorbent interacted with the polar parts of azimsulfuron and thiamethoxam through ionic interactions or hydrogen bonding, PSA/C18 led to the adsorption of azimsulfuron and thiamethoxam during the purification, as well as lower recovery [37]. Purification with C18 alone achieved recoveries above 70% for all pesticides and was expected to effectively remove impurities, such as sugars, fatty acids, and organic acids, from the samples [38].

The EN15662 extraction method and C18 purification were selected for optimal performance, as they met the Codex guidelines for recovery rates.

### 3.3. Final Extraction and Purification

**Figure 2:**
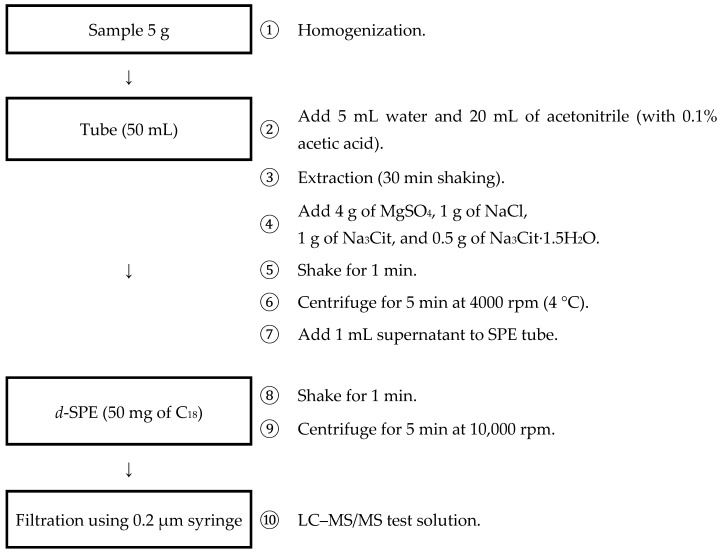
Analytical method for the extraction and purification of pesticide residues in fishery products. The figure illustrates the procedures and steps involved in the developed method for isolating and purifying pesticide residues from fishery products.

The optimized sample preparation procedure is detailed in Figure 2. Briefly, 5 g of the homogenized sample was added to a 50 mL Teflon tube, followed by 5 mL of water and 20 mL of acetonitrile containing 0.1 acetic acid. After shaking for 30 min, 4 g of MgSO_4_, 1 g of NaCl, 1 g of Na_3_Cit, and 1.5 g of Na_3_Cit·1.5H_2_O were added, and the mixture was shaken for an additional 30 min. The mixture was centrifuged at 4000 rpm for 5 min at 4 °C using a Megafuge 1.0 centrifuge (Thermo Fisher Scientific, Waltham, MA, USA). The acetonitrile and water layers were separated, and 1 mL of the supernatant (acetonitrile) was transferred to a dispersive solid-phase extraction (d-SPE) tube containing 50 mg of C18. After vortexing for 1 min and centrifugation at 10,000 rpm for 5 min, the supernatant was filtered through a 0.2 μm syringe filter to prepare the test solution for LC–MS/MS analysis.

### 3.4. Recovery, Linearity, LOD, LOQ, Matrix Effect

Method validation was conducted according to Codex guidelines [31] (Table 4). Recovery experiments were performed on representative fish species by spiking 24 pesticides at three different concentrations (LOQ, 10 × LOQ, 50 × LOQ) using the established method. Recovery rates for all the fortified samples were above 80%, meeting the Codex acceptance range (70–120%) for multiresidue analysis recovery.

Linearity was determined using standard calibration curves produced by LC–MS/MS. Matrix-matched calibration curves at seven different concentrations, including LOQ levels, were analyzed using LC–MS/MS. The concentration ranges for the calibration curves were 0.25, 0.75, 1.5, 3.75, 7.5, 12, 15 ng/g for 3 ng/g LOQ; 0.3, 1, 1.6, 2, 5, 10, 16, 20 ng/g for 4 ng/g LOQ; 0.4, 1.25, 2, 2.5, 6.25, 12.5, 20, 25 ng/g for 5 ng/g LOQ; 0.6, 1.75, 2.8, 3.5, 8.75, 17.5, 28, 35 ng/g for 7 ng/g LOQ; and 0.8, 2.5, 4, 5, 12.5, 25, 40, 50 ng/g for 10 ng/g LOQ. The coefficients of determination (R^2^) values for the 24 pesticides ranged from 0.9921 to 1.000, demonstrating excellent linearity in accordance with Codex guidelines (R^2^ > 0.98).

The LOD (S/N ≥ 3) ranged from 0.25 to 0.8 ng/g, and the LOQ (S/N ≥ 10) from 3 to 10 ng/g, depending on the pesticides and samples. Some pesticides, such as carbendazim, dichlorvos, and thiamethoxam, showed a higher LOQ (7–10 ng/g) owing to lower sensitivity of the LC–MS/MS. These LOQ results are sufficient for future applications in fishery product pesticide residue monitoring under PLS regulations.

To evaluate the matrix effect (ME) on pesticide ionization, matrix-matched calibration curves were compared to solvent-based curves. ME values between −20% and +20% indicated minimal interference from co-eluting substances, suggesting a weak matrix effect. ME values outside this range (>+50% or <−50%) indicated significant interference. Overall, most pesticides displayed low ME values, with only a few exceptions exceeding the ±20% range in specific samples, such as azinphos-methyl and carbofuran in eel, and tricyclazole in olive flounder and whiteleg shrimp.

### 3.5. Accuracy and Precision

Accuracy was evaluated based on recovery rates, whereas precision was assessed using the relative standard deviation (% RSD). Standard solutions of 24 pesticides at concentrations of LOQ, 10 × LOQ, and 50 × LOQ were spiked into untreated samples, and each sample was analyzed five times. Recoveries ranged from 71.7% to 106.3% at all concentration levels, meeting the Codex accuracy guidelines (70–120%). Precision (%RSD) ranged from 1.0% to 14.2% at LOQ, 0.6% to 9.0% at 10 × LOQ, and 0.4% to 6.9% at 50 × LOQ, all within the Codex requirement of ≤20%.

### 3.6. Monitoring Results for Pesticides

Based on the monitoring results, lufenuron, a benzoylurea insecticide, was detected at 10 ng/g in one out of the 300 eel samples analyzed. This pesticide is known for its non-systemic properties and inhibition of chitin synthesis [39], which effectively controls pests, such as diamondback moths. In South Korea, lufenuron is used to control pests in various agricultural products including sweet potatoes, peppers, blueberries, cherries, oranges, and grapes [40]. Pesticides with lufenuron as the active ingredient might have entered the aquatic environment and accumulated in eels. This finding underscores the necessity for the continuous monitoring of pesticide residues in fishery products to ensure food safety.

### 3.7. Risk Assessment for Fishery Product Consumption

**Figure 3:**
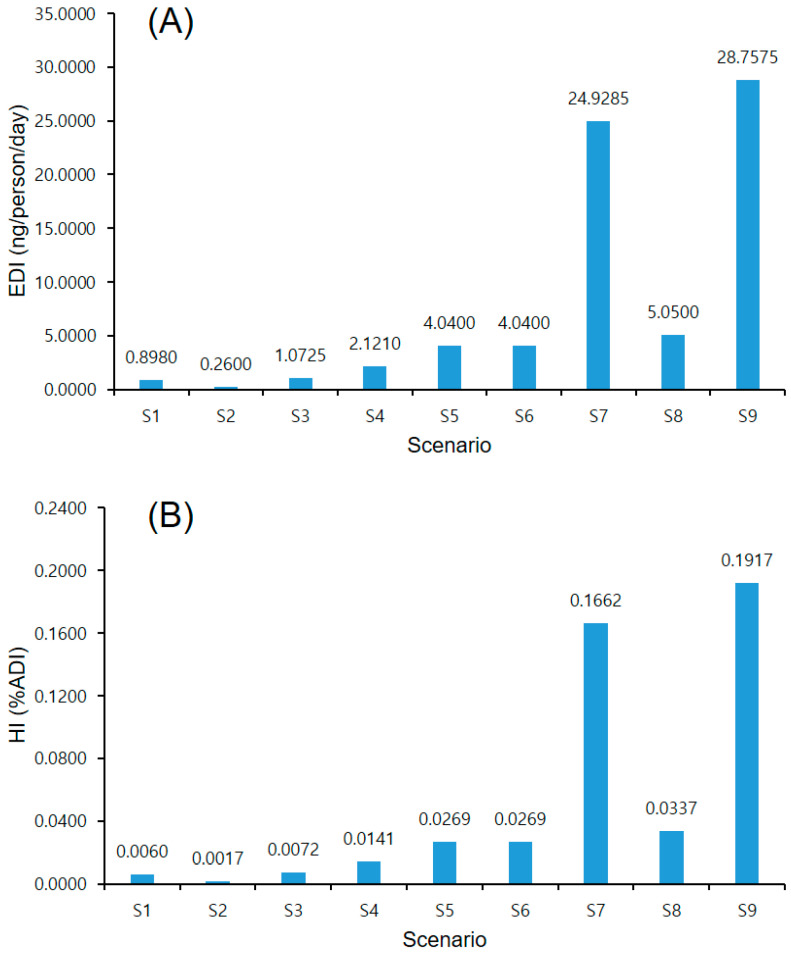
Assessment of dietary exposure to lufenuron in fishery products. (**A**) Estimated daily intake (EDI) and (**B**) health risk index (HI).

Risk assessment was based on pesticide residue detection and consumption data. The EDI and health risk index (%ADI) for various scenarios are shown in Figure 3. With an ADI of 0.015 mg/kg bw/day for lufenuron, the %ADI for average consumers ranged from 0.0017% to 0.0072 (scenarios 1–3). For extreme consumers of eel in which lufenuron was detected, the %ADI values (scenarios 4–6) ranged from 0.0141% to 0.0269%. The %ADI values for extreme consumers of different fishery product species (scenarios 7–9) ranged from 0.0337% to 0.1917%, with the highest value (scenario 9) still indicating a very low risk. Overall, the risk assessment across all the scenarios indicated that the detected pesticide residues posed a very low risk. According to the FAO and WHO guidelines, a %ADI below 10% signifies minimal risk [41]. Kim et al. [32] also reported low risk levels (0.00% to 1.07%) for farmed fish in South Korea. This study confirms that even a high consumption of contaminated fishery products poses a very low risk to human health. Nonetheless, the detection of pesticides that are not intended for fish highlights the need for continued monitoring and systematic management of pesticide residues.

## 4. Conclusions

We developed and validated an efficient pretreatment and analytical method for the detection of pesticide residues in fishery products. The EN15662 method achieved higher recovery rates than did the AOAC method, attaining a greater than 70% recovery rate for all the 24 pesticides analyzed. Although the combination of PSA and C18 resulted in lower recovery rates for some pesticides, the use of C18 alone consistently resulted in higher recovery rates for all pesticides. The newly developed analytical method complies with the Codex guidelines, demonstrating high accuracy, precision, and minimal matrix effects, which ensures minimal ionization interference from the sample matrix. This demonstrates that our method is suitable for the analysis of pesticide residues in various fishery products.

Risk assessment analysis indicated that all scenarios fell well below the 10% safety threshold set by the FAO and WHO, suggesting a very low potential risk to human health from the consumption of fishery products. However, the detection of pesticides not intended for use with fish underscores the importance of improving the safety management and monitoring of fishery products.

The method developed in this study provides essential baseline data for the safety assessment and management of fishery products and contributes to the safety of these products through continuous monitoring. Additionally, our findings support maintenance of the safety of fishery products in international trade and can serve as a valuable reference for establishing related regulations and standards.

## Figures and Tables

**Table 1:** Experimental conditions for LC–MS/MS analysis in multiple reaction monitoring mode.

No	Pesticide	Retention Time (min)	Precursor Ion (*m*/*z*)	Product Ion (*m*/*z*)	Collision Energy (eV)
1	Acetamiprid	5.448	223.1	126.1	−11
				55.9	−16
2	Azinphos-methyl	8.218	318.0	132.0	−16
				160.1	−7
3	Azoxystrobin	8.04	404.0	328.9	−26
				343.9	−20
4	Carbendazim	5.269	192.1	132.1	−25
				105.1	−37
5	Carbofuran	6.449	222.1	165.0	−6
				123.1	−11
6	Clothianidin	5.274	250.0	132.0	−16
				169.1	−13
7	Dichlorvos	6.487	238.0	109.1	−21
				220.9	−11
8	Dinotefuran	4.603	203.1	114.1	−13
				87.0	−16
9	Diuron (DCMU)	7.699	233.0	46.1	−17
				−8.0	39
10	Flubendiamide	9.605	683.0	408.0	−11
				274.0	−29
11	Hexaconazole	10.210	314.1	70.0	−22
				159.0	−30
12	Pyrimisulfan	7.421	420.1	370.1	−19
				388.1	−13
13	Terbuthylazine	8.652	230.1	174.0	−7
				68.1	−38
14	Thiacloprid	5.741	253.0	126.0	−11
				90.1	−39
15	Thiamethoxam	4.943	292.0	181.1	−22
				132.0	−22
16	Tricyclazole	6.006	190.1	136.0	−24
				109.0	−36
17	Azimsulfuron	7.42	424.9	182.1	−17
				156.0	−34
18	Trifroxystrobin	11.222	409.4	186.0	−20
				144.9	−45
19	Difenoconazole	10.816	405.9	250.9	−25
				337.0	−19
20	Fenobucarb	8.04	208.0	95.1	−14
				151.9	−10
21	Tebufenozide	9.454	353.0	133.0	−11
				297.1	−9
22	Lufenuron	12.291	510.8	158.1	−21
				141.1	−42
23	Indoxacarb	10.823	528.1	203.0	−38
				150.1	−22
24	Daimuron	8.717	269.2	151.2	−12
				119.2	−21

**Table 2:** Korean dietary consumption of 15 fish species.

Fish Species	Food Consumption (g/Person/Day) in KNHANES ^a^ (2017–2021)	
	Mean	Extreme (99th Percentile)
Abalone	0.6000	13.8000
Carp	0.4800	12.1200
Chinese muddy loach	0.9600	43.6800
Crucian carp	0.4800	12.1200
Eel	1.3200	24.2400
Far eastern catfish	0.4800	12.1200
Flathead mullet	0.4800	12.1200
Korean rockfish	1.2000	31.2000
Mirror carp	0.4800	12.1200
Olive flounder	1.3500	48.4500
Rainbow trout	0.4800	12.1200
Red seabream	0.4800	12.1200
Sea bass	0.4800	12.1200
Starry flounder	0.4800	12.1200
Whiteleg shrimp	1.8000	50.4000

^a^ Korea National Health and Nutrition Examination Survey.

**Table 3:** Comparison of pesticide recovery rates for different extraction and clean-up methods.

Pesticide	Recoveries (Mean ± Standard Deviation) (%)			
	Extraction Method		Clean-Up Materials	
	EN 15662 ^a^	AOAC ^b^	C18 50 mg	PSA 50 mg + C18 50 mg
Acetamiprid	87.9 ± 4.6	77.8 ± 6.7	75.9 ± 0.6	75.4 ± 1.3
Azinphos-methyl	84.0 ± 1.8	68.3 ± 7.4	75.4 ± 4.8	74.2 ± 3.4
Azoxystrobin	80.8 ± 1.6	90.9 ± 4.2	88.1 ± 2.5	80.5 ± 2.2
Carbendazim	89.7 ± 3.1	49.9 ± 2.6	86.2 ± 2.8	75.0 ± 2.7
Carbofuran	69.1 ± 4.2	70.5 ± 7.7	81.8 ± 6.5	80.5 ± 1.9
Clothianidin	72.1 ± 3.3	74.7 ± 6.8	80.8 ± 1.5	84.2 ± 6.0
Dichlorvos	63.5 ± 6.4	54.4 ± 7.0	77.2 ± 3.0	69.7 ± 2.0
Dinotefuran	72.7 ± 1.9	64.3 ± 6.0	77.9 ± 3.8	92.8 ± 3.4
Diuron	70.3 ± 4.3	68.0 ± 5.6	80.2 ± 0.3	75.4 ± 2.7
Flubendiamide	89.3 ± 4.8	55.2 ± 1.6	80.2 ± 2.5	73.4 ± 4.9
Hexaconazole	70.4 ± 5.1	65.2 ± 5.1	81.9 ± 1.7	77.6 ± 2.8
Pyrimisulfan	86.5 ± 4.0	77.9 ± 4.4	83.6 ± 4.6	265.7 ± 26.1
Terbuthylazine	76.6 ± 3.5	57.6 ± 3.2	81.4 ± 4.0	76.6 ± 1.9
Thiacloprid	81.9 ± 5.9	75.4 ± 7.2	80.3 ± 3.7	76.8 ± 2.9
Thiamethoxam	78.7 ± 6.5	30.0 ± 3.1	86.2 ± 2.8	54.8 ± 3.4
Tricyclazole	78.4 ± 3.2	60.8 ± 4.7	85.4 ± 3.7	77.5 ± 2.4
Azimsulfuron	77.6 ± 0.3	60.8 ± 3.1	83.3 ± 0.5	−3.5 ± 4.9
Trifroxystrobin	74.6 ± 4.2	59.5 ± 1.5	82.2 ± 1.8	80.6 ± 1.9
Difenoconazole	76.9 ± 3.8	48.4 ± 5.0	73.6 ± 4.5	79.5 ± 2.0
Fenobucarb	76.5 ± 5.9	74.6 ± 2.8	86.4 ± 3.1	80.7 ± 3.4
Tebufenozide	76.7 ± 3.7	90.4 ± 3.4	76.0 ± 2.7	76.6 ± 0.5
Lufenuron	96.7 ± 5.0	74.5 ± 3.9	77.5 ± 5.6	80.0 ± 3.6
Indoxacarb	79.4 ± 2.8	67.8 ± 3.6	79.1 ± 3.3	98.4 ± 3.2
Daimuron	80.5 ± 1.7	36.5 ± 2.2	87.8 ± 3.4	80.7 ± 1.3

^a^ 20 mL of acetonitrile containing 0.1% of acetic acid, 4 g of MgSO_4_, 1 g of NaCl, and 1.5 g of Na_3_Cit were used in the EN 15662 method for the extraction of pesticides. ^b^ 20 mL of acetonitrile containing 0.1% of acetic acid, 6 g of MgSO_4_, and 1.5 g of sodium acetate were used in the AOAC method for the extraction of pesticides.

**Table 4:** Linearity, matrix effect, limit of detection (LOD), limit of quantification (LOQ), recovery, and precision of multiclass pesticides.

Pesticides	Matrix	Linearity (R2)	Matrix Effect	LOD (ng/g Wet Weight)	LOQ (ng/g Wet Weight)	Relative Recovery (%)			RSD ^a^ (%)		
						LOQ	10 × LOQ	50 × LOQ	LOQ	10 × LOQ	50 × LOQ
Acetamiprid	Eel	0.9999	16.80	0.4	5	88.8	90.1	92.7	2.2	1.4	2.8
	Flatfish	0.9993	−2.73	0.4	5	84.4	78.9	93.2	1.5	9.0	1.1
	Avalone	1.0000	−5.60	0.25	3	88.9	90.1	87.9	2.8	1.1	2.9
	Shrimp	0.9997	−9.55	0.6	7	90.8	92.8	93.7	1.5	1.4	2.1
Azinphos-methyl	Eel	0.9995	24.87	0.25	3	89.7	89.8	90.7	12.1	1.9	2.5
	Flatfish	0.9990	5.85	0.25	3	84.0	86.6	93.1	6.1	3.1	4.6
	Avalone	0.9989	−1.87	0.3	4	85.1	91.7	90.5	7.3	2.5	5.0
	Shrimp	0.9998	0.77	0.4	5	95.4	90.0	92.7	4.1	2.3	1.0
Azoxystrobin	Eel	0.9998	19.44	0.3	4	89.4	90.5	92.1	2.2	1.1	4.1
	Flatfish	0.9994	−0.002	0.25	3	83.9	87.9	94.6	5.7	1.4	2.1
	Avalone	0.9998	−4.59	0.25	3	90.4	91.4	91.4	7.1	3.3	2.9
	Shrimp	0.9995	−5.99	0.3	4	85.8	93.8	94.0	5.7	1.5	3.3
Carbendazim	Eel	0.9995	15.06	0.3	4	86.7	85.8	88.6	4.5	3.4	4.9
	Flatfish	0.9989	−8.80	0.6	7	80.2	79.0	87.7	4.1	3.5	2.1
	Avalone	0.9999	−11.90	0.4	5	86.7	88.3	85.7	3.9	1.9	4.7
	Shrimp	0.9992	−15.11	0.25	3	87.6	91.0	89.1	8.4	3.3	5.2
Carbofuran	Eel	0.9996	24.36	0.25	3	91.8	88.8	92.7	4.7	1.2	3.8
	Flatfish	0.9994	0.83	0.25	3	87.2	87.9	93.7	4.2	1.4	1.4
	Avalone	0.9998	−0.31	0.3	4	93.4	89.5	88.4	5.0	0.7	2.9
	Shrimp	0.9995	−4.04	0.4	5	90.0	92.3	94.1	5.2	2.1	2.2
Clothianidin	Eel	0.9994	23.75	0.25	3	95.4	91.0	93.9	5.1	4.2	4.0
	Flatfish	0.9992	−2.45	0.3	4	75.2	83.4	91.0	11.2	1.9	2.9
	Avalone	0.9997	−6.90	0.3	4	85.8	88.6	85.9	10.5	3.7	2.4
	Shrimp	0.9983	−6.53	0.4	5	84.2	92.5	91.5	2.8	2.7	2.0
Dichlorvos	Eel	0.9996	15.87	0.4	5	84.0	88.1	92.7	7.1	2.1	1.7
	Flatfish	0.9988	−4.16	0.4	5	82.7	85.1	93.7	8.5	1.5	3.2
	Avalone	0.9999	−6.48	0.8	10	88.9	90.8	91.4	5.6	2.4	2.7
	Shrimp	0.9993	−9.03	0.8	10	71.7	89.7	92.6	9.9	1.6	2.3
Dinotefuran	Eel	0.9998	5.45	0.3	4	87.1	88.9	91.4	5.3	1.6	1.6
	Flatfish	0.9990	−1.80	0.3	4	82.1	84.8	90.2	3.9	2.5	1.6
	Avalone	0.9999	−9.93	0.4	5	89.2	88.7	87.1	6.5	1.3	3.0
	Shrimp	0.9996	25.45	0.4	5	88.1	89.5	91.9	1.6	2.7	2.0
Diuron	Eel	0.9996	27.79	0.4	5	91.5	89.0	92.0	4.3	1.6	1.6
	Flatfish	0.9991	−1.96	0.25	3	84.8	83.5	92.0	5.7	1.8	0.5
	Avalone	0.9999	−3.49	0.3	4	93.2	89.3	89.6	3.6	1.9	2.1
	Shrimp	0.9991	−3.49	0.3	4	93.2	89.3	89.6	3.6	1.9	2.1
Flubendiamide	Eel	0.9991	21.29	0.25	3	88.3	87.3	88.5	6.2	2.7	5.9
	Flatfish	0.9976	7.12	0.4	4	92.8	88.8	95.1	5.6	3.1	3.8
	Avalone	0.9995	2.04	0.4	4	86.2	93.4	90.7	8.1	1.5	4.3
	Shrimp	0.9965	0.89	0.25	3	95.3	92.8	93.7	10.7	4.1	4.9
Hexaconazole	Eel	0.9921	−1.77	0.25	3	88.3	93.9	97.1	4.9	1.2	3.8
	Flatfish	0.9988	−2.58	0.25	3	83.7	84.9	89.3	5.8	3.2	2.9
	Avalone	0.9996	−8.25	0.25	3	77.6	90.5	87.9	13.2	2.3	3.1
	Shrimp	0.9990	−7.47	0.25	3	82.9	91.4	92.9	5.8	2.9	5.2
Pyrimisulfan	Eel	0.9998	13.62	0.4	5	90.3	89.0	92.2	1.9	1.2	3.8
	Flatfish	0.9990	6.35	0.3	4	87.6	86.6	93.4	2.0	1.2	1.1
	Avalone	0.9996	1.08	0.25	3	90.2	90.2	89.2	2.7	1.1	2.5
	Shrimp	0.9997	3.16	0.3	4	88.6	90.9	92.8	1.2	1.8	2.1
Terbuthylazine	Eel	0.9995	28.46	0.4	5	88.8	86.7	90.1	2.6	0.6	3.7
	Flatfish	0.9994	−1.35	0.25	3	87.0	85.7	90.9	5.1	1.8	1.5
	Avalone	0.9999	−5.93	0.3	4	90.1	91.6	89.7	4.4	1.3	2.9
	Shrimp	0.9994	−4.81	0.3	4	82.4	92.1	91.5	6.2	3.5	3.8
Thiacloprid	Eel	0.9999	20.48	0.4	5	84.8	90.3	91.9	2.8	1.2	4.2
	Flatfish	0.9995	0.82	0.4	5	85.0	86.0	92.5	2.3	1.9	1.1
	Avalone	0.9939	−2.12	0.3	4	88.4	89.9	86.7	2.8	1.7	0.4
	Shrimp	0.9994	−6.37	0.6	7	86.1	93.0	93.0	2.4	1.3	1.4
Thiamethoxam	Eel	0.9997	16.73	0.25	3	93.4	90.2	93.9	7.7	1.1	2.7
	Flatfish	0.9995	−11.66	0.6	7	81.2	82.5	91.9	2.8	2.6	1.8
	Avalone	1.0000	−14.10	0.3	4	90.6	89.0	87.5	5.1	1.1	2.3
	Shrimp	0.9991	−6.82	0.4	5	85.0	92.1	94.2	4.5	2.3	2.4
Tricyclazole	Eel	0.9997	−0.08	0.25	3	90.1	90.7	101.3	3.3	0.8	2.4
	Flatfish	0.9993	−20.33	0.4	5	86.3	87.3	106.3	1.0	2.0	1.9
	Avalone	0.9998	−19.23	0.4	5	85.6	88.9	93.9	5.1	0.8	2.2
	Shrimp	0.9979	−26.81	0.3	4	90.6	95.4	101.0	3.6	1.1	1.7
Azimsulfuron	Eel	0.9998	15.92	0.3	4	87.5	88.2	91.1	3.1	1.5	2.2
	Flatfish	0.9993	6.15	0.3	4	83.0	83.4	90.5	3.6	1.7	1.9
	Avalone	0.9997	1.80	0.3	4	85.8	90.0	89.2	3.7	2.1	2.1
	Shrimp	0.9997	4.49	0.25	3	85.1	88.2	90.9	3.8	1.7	2.8
Trifroxystrobin	Eel	0.9990	27.81	0.4	5	96.8	90.9	92.7	4.5	3.0	3.8
	Flatfish	0.9984	−3.56	0.25	3	90.4	90.5	95.8	2.7	2.3	1.8
	Avalone	0.9998	−3.16	0.25	3	87.8	90.5	88.7	4.3	2.1	2.1
	Shrimp	0.9986	−10.76	0.25	3	87.1	94.6	100.5	7.8	2.1	1.5
Difenoconazole	Eel	0.9993	16.22	0.3	4	86.3	88.7	92.5	1.8	1.4	2.2
	Flatfish	0.9990	1.25	0.3	4	83.0	86.1	91.9	3.4	0.7	1.0
	Avalone	1.0000	−5.08	0.25	3	87.6	91.8	90.5	5.8	0.8	2.5
	Shrimp	0.9996	−4.78	0.25	3	86.3	92.3	91.7	4.8	1.5	2.4
Fenobucarb	Eel	0.9997	18.71	0.3	4	87.6	88.9	92.2	3.9	1.8	2.2
	Flatfish	0.9990	−0.45	0.3	4	81.7	86.0	91.7	7.4	1.2	2.3
	Avalone	0.9997	−4.37	0.3	4	88.7	92.2	89.1	5.3	1.8	2.9
	Shrimp	0.9994	−2.13	0.25	3	90.1	93.7	93.1	3.1	1.7	2.0
Tebufenozide	Eel	0.9998	22.51	0.25	3	92.1	90.6	93.9	2.8	1.8	3.9
	Flatfish	0.9993	0.71	0.4	5	83.1	86.8	94.4	3.6	1.7	1.6
	Avalone	0.9998	−3.82	0.3	4	84.2	91.5	89.0	4.6	1.7	1.9
	Shrimp	0.9989	−3.61	0.4	5	90.5	91.6	93.8	3.7	2.1	4.3
Lufenuron	Eel	0.9993	20.25	0.4	5	87.6	86.6	87.2	5.3	2.4	6.7
	Flatfish	0.9997	−10.19	0.4	5	78.8	91.9	91.6	12.0	7.0	3.1
	Avalone	0.9997	−17.85	0.3	4	80.4	91.9	84.1	14.2	2.2	6.9
	Shrimp	0.9992	−3.16	0.25	3	93.7	94.0	95.0	12.9	2.5	4.9
Indoxacarb	Eel	0.9997	15.40	0.4	5	90.8	89.2	92.6	2.2	2.4	5.1
	Flatfish	0.9976	5.46	0.3	4	81.6	88.7	93.7	12.5	2.6	2.7
	Avalone	0.9997	−1.23	0.4	5	94.1	90.9	90.5	7.7	2.0	1.2
	Shrimp	0.9988	−2.55	0.3	4	89.2	90.8	92.8	7.2	2.3	6.6
Daimuron	Eel	0.9998	16.93	0.3	4	87.2	88.9	91.4	1.3	1.5	3.6
	Flatfish	0.9993	1.61	0.3	4	87.2	86.7	93.1	1.7	0.8	0.9
	Avalone	0.9999	−2.36	0.25	3	88.7	91.4	90.4	1.6	0.9	2.3
	Shrimp	0.9997	−3.47	0.25	3	89.1	91.6	93.3	3.0	1.6	2.7

^a^ Relative standard deviation of repeatability (intra-day tests).

## Data Availability

The data presented in this study are available upon request from the corresponding author owing to legal restrictions.
